# TPPU, a soluble epoxide hydrolase inhibitor, mitigates Aβ-induced neurotoxicity via dual modulation of mitochondrial stabilization and JNK suppression

**DOI:** 10.1016/j.bbrep.2026.102806

**Published:** 2026-10-12

**Authors:** Jingfang Song, Ailan He, Shuyu Li, Qiong Wu

**Affiliations:** a Department of General Surgery, Wuyi Hospital of Traditional Chinese Medicine, Jiangmen, 529000, China; b Department of Clinical Medicine, Guangdong Jiangmen Chinese Medicine College, Jiangmen, 529000, China; c Department of Basic Medicine, Guangdong Jiangmen Chinese Medicine College, Jiangmen, 529000, China

**Keywords:** Alzheimer's disease, Soluble epoxide hydrolase, TPPU, Apoptosis, JNK signaling

## Abstract

Alzheimer's disease (AD) is a neurodegenerative disorder characterized by progressive cognitive decline, primarily driven by β-amyloid (Aβ) aggregation and neuronal apoptosis. This study aims to investigate the neuroprotective effects of soluble epoxide hydrolase (sEH) inhibition against Aβ-induced cytotoxicity in PC-12 cells, focusing on cell viability, apoptosis, and the c-Jun N-terminal kinase (JNK) signaling pathway. Western blotting and fluorometric assays revealed elevated sEH expression and activity in Aβ1-42-treated PC-12 cells compared to untreated controls. Treatment with N-[1-(1-Oxopropyl)-4-piperidinyl]-N'-[4-(trifluoromethoxy)phenyl]urea (TPPU), a potent sEH inhibitor, significantly attenuated Aβ-induced apoptosis in PC-12 cells and enhanced cell viability. These findings were corroborated by CCK-8 assays and flow cytometry analyses. Furthermore, TPPU reversed Aβ-induced cleaved Caspase-3 dysregulation and restored mitochondrial membrane potential. Notably, exogenous administration of epoxyeicosatrienoic acids partially mimicked the protective effects of TPPU, primarily by preserving mitochondrial function. Western blot analysis confirmed that TPPU pretreatment suppressed the phosphorylation of JNK, thereby contributing to its anti-apoptotic effects. Collectively, these findings demonstrate that TPPU attenuates Aβ-induced neurotoxicity via dual mechanisms—restoring mitochondrial function and suppressing JNK activation—highlighting its potential as a therapeutic agent for AD.

## Highlights

• sEH inhibition alleviates Aβ-induced neurotoxicity in PC-12 cells.
• TPPU protects neurons by stabilizing mitochondrial membrane potential and suppressing JNK activation.
• Exogenous EETs partially recapitulate the neuroprotective effects of TPPU.
• This study is the first to link sEH with Aβ-specific JNK dysregulation.

## Graphical abstract

**Figure:**
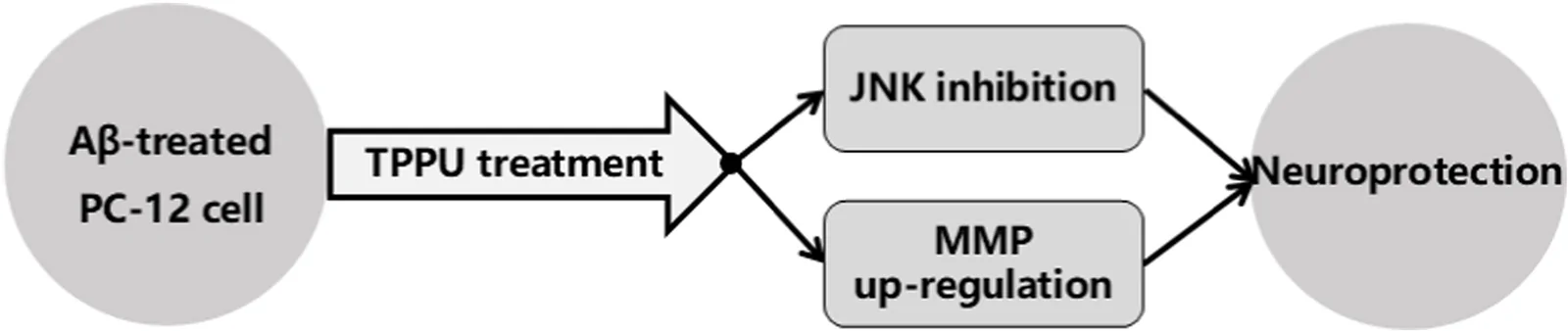

## 1 Introduction

At present, Alzheimer's disease (AD) is considered one of the leading neurodegenerative disorders, contributing to global neuronal degeneration. Notably, this disease has affected millions of individuals, imposing a significant socioeconomic burden on healthcare systems [1,2]. Clinically, AD is characterized by progressive behavioral changes, cognitive decline and memory loss, ultimately leading to functional disability and death. Despite this notable impact on patients, current therapeutic strategies that inhibit disease progression remain limited [3,4]. Considering global aging populations and medical advancements, novel strategies are urgently required to address challenges in the management of this condition [5].

The specific molecular mechanisms underlying AD progression involve multiple pathological cascades, including Aβ plaque deposition, tau hyperphosphorylation and neuroinflammation [6]. These factors disrupt physiology, leading to structural, functional and biochemical changes in AD neurons. The neurotoxicity of Aβ oligomers is a critical component within the AD pathological cascade [7]. These oligomers mediate neuronal damage through multiple mechanisms, including synaptic dysfunction and impaired plasticity [8], dysregulation of calcium homeostasis [9], mitochondrial dysfunction [10] and neuroinflammation [11]. Aβ is a key factor in AD development, triggering neurotoxic cascades, such as free radical production, mitochondrial dysfunction and inflammation [2,6,12,13]. Therefore, understanding the molecular pathways that modify Aβ-induced toxicity is crucial for the development of novel strategies that inhibit disease progression.

Soluble epoxide hydrolase (sEH) is a key node within the cytochrome P450 (CYP450)-epoxygenase pathway of arachidonic acid metabolism [14]. Notably, sEH hydrolyses epoxyeicosatrienoic acids (EETs) and bioactive lipids, regulating physiological processes, such as vasodilation, inflammation, anti-apoptotic actions and cell survival [15]. EETs protect neurons from oxidative stress and inflammation, which are two key hallmarks of AD. The protective role of EETs underscores their potential as therapeutic targets for mitigating the neurodegenerative processes associated with AD [16]. EETs are catalyzed by sEH into their inactive dihydroxyeicosatrienoic acid derivatives [17]. This modulation affects their bioavailability and downstream effects. Results of a previous study revealed that sEH plays a pivotal role in regulating neuroinflammation and oxidative stress, which are key factors in AD progression [18].

Ghosh et al. previously reported that sEH is upregulated in human brains with AD, and further demonstrated the role of this enzyme in exacerbating neuroinflammation in transgenic mice with five familial Alzheimer's disease (5xFAD) mice [19]. Notably, suppression of sEH is used as a therapeutic strategy to restore the beneficial effects of EETs, while decreasing the neurotoxic effects of Aβ [20]. Thus, sEH inhibition may exhibit potential in various neurodegenerative disease models [21]. The present study aimed to investigate the potential mechanisms by which sEH inhibition mitigates Aβ-induced neuronal damage *in vitro*, focusing on mitochondrial dysfunction and JNK signaling pathways. To the best of our knowledge, the present study is the first to explore these mechanisms in the context of sEH-AD therapeutics.

Notably, c-Jun N-terminal kinase (JNK) is member of the mitogen-activated protein kinase (MAPK) family that is pivotal in cellular stress responses, inflammation and apoptosis [22]. In AD, Aβ plaque accumulation activates several signaling pathways, such as JNK, causing neuroinflammation and neuronal damage [23]. Oxidative stress and inflammatory cytokines activate JNK, which are key contributors to AD pathology [24,25]. Persistent JNK activation, particularly in its phosphorylated form (p-JNK), becomes maladaptive, leading to neuronal apoptosis and disease progression [23]. JNK influences apoptosis through the regulation of gene transcription, directly acting on mitochondrial proteins, thereby modulating mitochondrial function and the apoptotic process [26,27]. In AD, chronic JNK activation, particularly via p-JNK, accelerates neurodegeneration [[23], [24], [25]]. JNKs play a central role in microglia activation, triggering neuroinflammation and the production of pro-inflammatory cytokines, which exacerbate neuronal damage [28,29]. Results of a previous *in vivo* study suggested that JNKs exert systemic effects on various cell types, including neurons, microglia and astrocytes [30]. JNK contributes to both neurodegeneration and neuroprotective mechanisms [29,31]; however, this protein predominantly exerts pro-apoptotic effects in chronic AD. Moreover, results of previous studies demonstrated that the JNK signaling pathway is intimately involved in the pathological progression of AD, influencing processes such as Aβ production, tau protein hyperphosphorylation, neuroinflammation and apoptosis [24,25].

Notably, a recent study by Yi et al. (2021) reported that TPPU, a potent sEH inhibitor, protects against ischemic stroke-induced neuronal damage by suppressing the JNK/p38 MAPK-mediated mitochondrial apoptosis pathway [32]. However, that study was conducted in an ischemic model, not in Alzheimer's disease. Whether TPPU exerts similar neuroprotective effects against Aβ-induced toxicity, and specifically through concurrent restoration of mitochondrial membrane potential and suppression of JNK activation, remains unknown.

This study investigated the effects of soluble epoxide hydrolase (sEH) inhibition on Aβ-induced cytotoxicity in PC-12 cells. We focused on three objectives: (1) determining whether the sEH inhibitor TPPU attenuates Aβ-induced deficits in cell viability and apoptosis; (2) evaluating TPPU's regulatory role in JNK pathway activation under Aβ toxicity; and (3) exploring the mechanistic link between sEH inhibition and mitochondrial membrane potential (MMP) stabilization. These aims were addressed using integrated methodologies, including CCK-8 assays, flow cytometry, Western blotting, and JC-1-based MMP quantification.

## 2 Materials and methods

### 2.1 General materials

PC-12 cells (cat. no. SNL-124; SUNNCELL) were authenticated via STR profiling and confirmed to be mycoplasma-free. Cells were cultured in Dulbecco's Modified Eagle's MediμM (DMEM; cat. no. SH30243.01; Hyclone; Cytiva) supplemented with fetal bovine serμM (FBS; cat. no. 11011-8611; Zhejiang Tianhang Biotechnology Co., Ltd.) and penicillin/streptomycin (cat. no. C0222; Beyotime Institute of Biotechnology). TPPU (cat. no. SML0750; Sigma-Aldrich; Merck KGaA) and Aβ1-42 (cat. no. 131602-53-4; Sigma-Aldrich; Merck KGaA) were used in the present study. EETs, including 5,6-EET (cat. no. 50211; Cayman Chemical Company), 8,9-EET (cat. no. 50351; Cayman Chemical Company), 11,12-EET (cat. no. 50511; Cayman Chemical Company) and 14,15-EET (cat. no. 50651; Cayman Chemical Company) were also used in the present study. Hoechst 33342 (cat. no. C1026; Beyotime Institute of Biotechnology) was used for nuclear staining. The JNK inhibitor, SP600125 (cat. no. S1876; Beyotime Institute of Biotechnology), was used to inhibit the JNK signaling pathway. Cell Counting Kit-8 (CCK-8; cat. no. CK04; Dojindo Molecular Technologies, Inc.) was used for cell viability assays.

Primary antibodies used in the present study were as follows: Anti-Ephx2 (cat. no. ab155280; Abcam), anti-cleaved Caspase-3 (cat. no. 9661T; Cell Signaling Biotechnology, Inc.), anti-JNK (cat. no. J4750; Sigma-Aldrich; Merck KgaA), p-JNK (cat. no. sc-81502; Santa Cruz Biotechnology, Inc.) and β-actin (cat. no. M01263-4; Wuhan Boster Biological Technology, Ltd.). Secondary antibodies included HRP-labeled goat anti-mouse IgG (H + L; cat. no. A0216; Beyotime Institute of Biotechnology) and HRP-labeled goat anti-rabbit IgG (H + L; cat. no. A0208; Beyotime Institute of Biotechnology). Kits included the Caspase-3 Activity and Mitochondrial Membrane Potential Detection kit (cat. no. C1073 M; Beyotime Institute of Biotechnology), the Annexin V-FITC Apoptosis Detection kit (cat. no. C1062; Beyotime Institute of Biotechnology) and the Soluble Epoxide Hydrolase Cell-Based Assay kit (cat. no. 600090; Cayman Chemical Company).

Working solutions of TPPU were prepared by dissolving the compound in pure polyethylene glycol 400 (PEG 400) and diluting with distilled water to achieve a final concentration of 40%. EETs were dried under nitrogen gas and reconstituted in 40% PEG 400, while Aβ was dissolved in DMSO (cat. no. D2650; Sigma-Aldrich; Merck KGgA) for experimental use. All treatments contained 0.1% DMSO and/or 0.4% PEG 400, with matched vehicle controls.

### 2.2 Cell culture and treatment

PC-12 cells were maintained in culture using DMEM enriched with 10% heat-inactivated FBS and 1% penicillin/streptomycin. Cells were incubated at 37°C in an environment containing 5% CO_2_. To induce cytotoxicity, cells were exposed to oligomeric Aβ1-42 (50 μM) for 24 h. Prior to Aβ1-42 treatment, selected groups were pre-treated with the sEH inhibitor, TPPU (25 μM) at 37°C, for 2 h to assess the potential neuroprotective effects. Based on results of the CCK-8 EC80 assay, 25 μM, the lowest concentration that restored cell viability to >80% of the control level, was selected for all subsequent experiments (81.0% viability recovery; Fig. 2A).

### 2.3 CCK-8 assay

Cells were seeded into 96-well plates. Following the treatment phase, 10 μl of CCK-8 reagent was added to each well. Following incubation for 2 h, the absorbance was measured at 450 nm to determine cell viability.

### 2.4 Hoechst 33342 staining

Cells were cultured on coverslips in a 24-well plate and treated as previously described. Following washing with PBS, cells were stained with Hoechst 33342 (10 μg/ml) at 37°C for 30 min, and images were obtained using a fluorescence microscope.

### 2.5 MMP assay

Cells were treated and subsequently incubated with JC-1 staining solution (10 μg/ml) for 20-30 min at 37°C. Following incubation, cells were washed with PBS to remove excess dye and subsequently analyzed using a fluorescence microscope. JC-1 aggregates were detected using red fluorescence, and this was indicative of a high MMP. By contrast, JC-1 monomers were detected using green fluorescence, and these were indicative of a low MMP.

### 2.6 Annexin V-FITC apoptosis assay

PC-12 cells were seeded in six-well plates at 3 × 10^5^ cells/well, treated as previously described and detached using EDTA-free trypsin to retain apoptotic cells. Following washing with ice-cold PBS, cells were resuspended in 195 μl of binding buffer, and stained with 5 μl of Annexin V-FITC and 10 μl of PI for 15 min at 37°C in the dark. Samples were analyzed using a CytoFLEX flow cytometer (50,000 events) and apoptotic populations were quantified via CytExpert software (Beckman). Cells were classified as follows: i) Viable cells, Annexin V^−^/PI^−^; ii) early apoptotic cells, Annexin V^+^/PI^−^; iii) late apoptotic/necrotic cells, annexin V^+^/PI^+^; and iv) necrotic cells, Annexin V^−^/PI^+^.

### 2.7 sEH enzyme activity assay

PC-12 cells were cultured in 96-well plates. When 80% confluence was reached, cells were treated with 50 μM Aβ1-42 for 24 h. Following treatment, sEH activity was quantified using the Soluble Epoxide Hydrolase Cell-Based Assay kit, according to the manufacturer's protocol.

### 2.8 Western blot analysis

Cells were lysed in ice-cold RIPA buffer (50 mM Tris-HCl pH 7.4, 150 mM NaCl, 1% NP-40, 0.5% sodiμM deoxycholate, 0.1% SDS) supplemented with 1 mM PMSF. Lysates were centrifuged at 12,000 × g for 15 min at 4°C, and supernatants collected. Protein lysates (20 μg) were separated via SDS-PAGE on 10% gels and transferred to PVDF membranes. Following blocking with 5% skimmed milk for 1 h at 37°C, membranes were probed with primary antibodies against Ephx2 (1:10,000), JNK (1:1000), p-JNK (1:500), cleaved Caspase-3 (1:1000) and β-actin (1:5000) overnight at 4°C. Following primary incubation, membranes were incubated with HRP-labeled secondary antibodies (1:5000) for 1 h, and immunoreactive bands were detected using enhanced chemilμMinescence.

### 2.9 Statistical analysis

Results are presented as the mean ± SEM. Differences between groups were assessed using one-way ANOVA followed by LSD for post-hoc analysis. Statistical analysis was performed using SPSS software (version, 23.0; IBM Corp.). P < 0.05 was considered to indicate a statistically significant difference.

## 3 Results

### 3.1 Aβ induces sEH expression and enhances its activity in PC-12 cells

Exposure to Aβ1-42 significantly upregulated both sEH protein expression and enzymatic activity. Following 24 h of treatment with Aβ1-42, sEH levels and activity were quantified via Western blot analysis (Fig. 1A and B) and fluorometric assays (Fig. 1C), respectively. Aβ1-42 treatment increased sEH protein expression by 1.6-fold (η^2^ = 0.70; P = 0.01) and enzymatic activity by 1.5-fold (η^2^ = 0.73; P < 0.001) compared with controls (Fig. 1C), suggesting that Aβ toxicity directly modulated sEH regulation. Notably, sEH activity was measured using a commercial kit; therefore, contributions from non-specific esterases may have gone undetected.

**Fig. 1:**
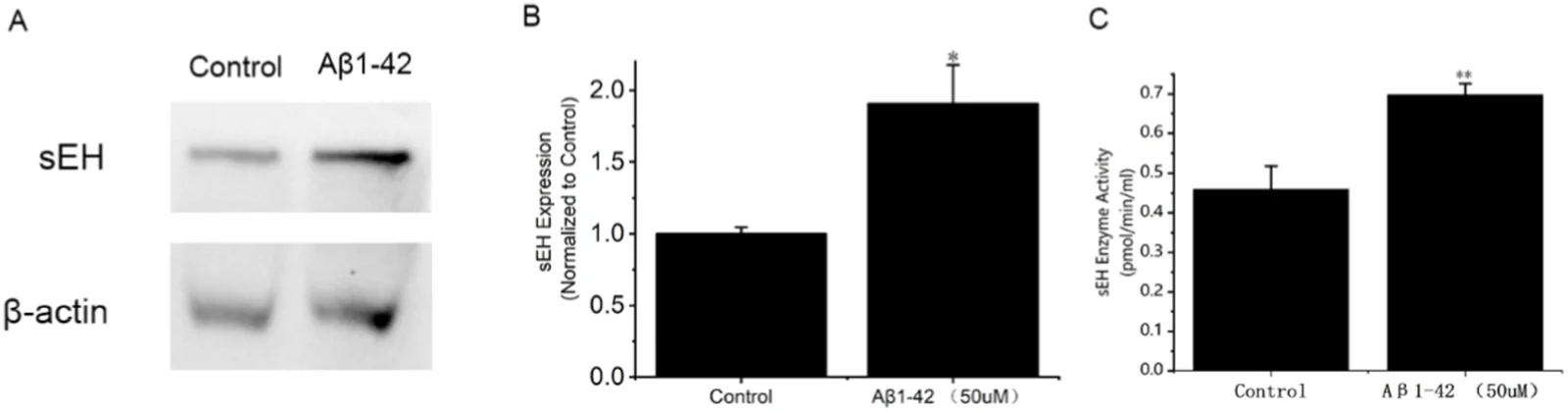
The impact of Aβ1-42 on sEH expression and activity in PC-12 cells. A. PC-12 cells were treated with 50 μM Aβ1-42 for 24 h, followed by Western blot analysis to evaluate sEH expression. B. The quantification of sEH expression through Western blotting (n = 4). C. The measurement of sEH enzyme activity (n = 6). Data = mean ± SEM. *p < 0.05 compared to control. ***p < 0.001 compared to control.

**Fig. 2:**
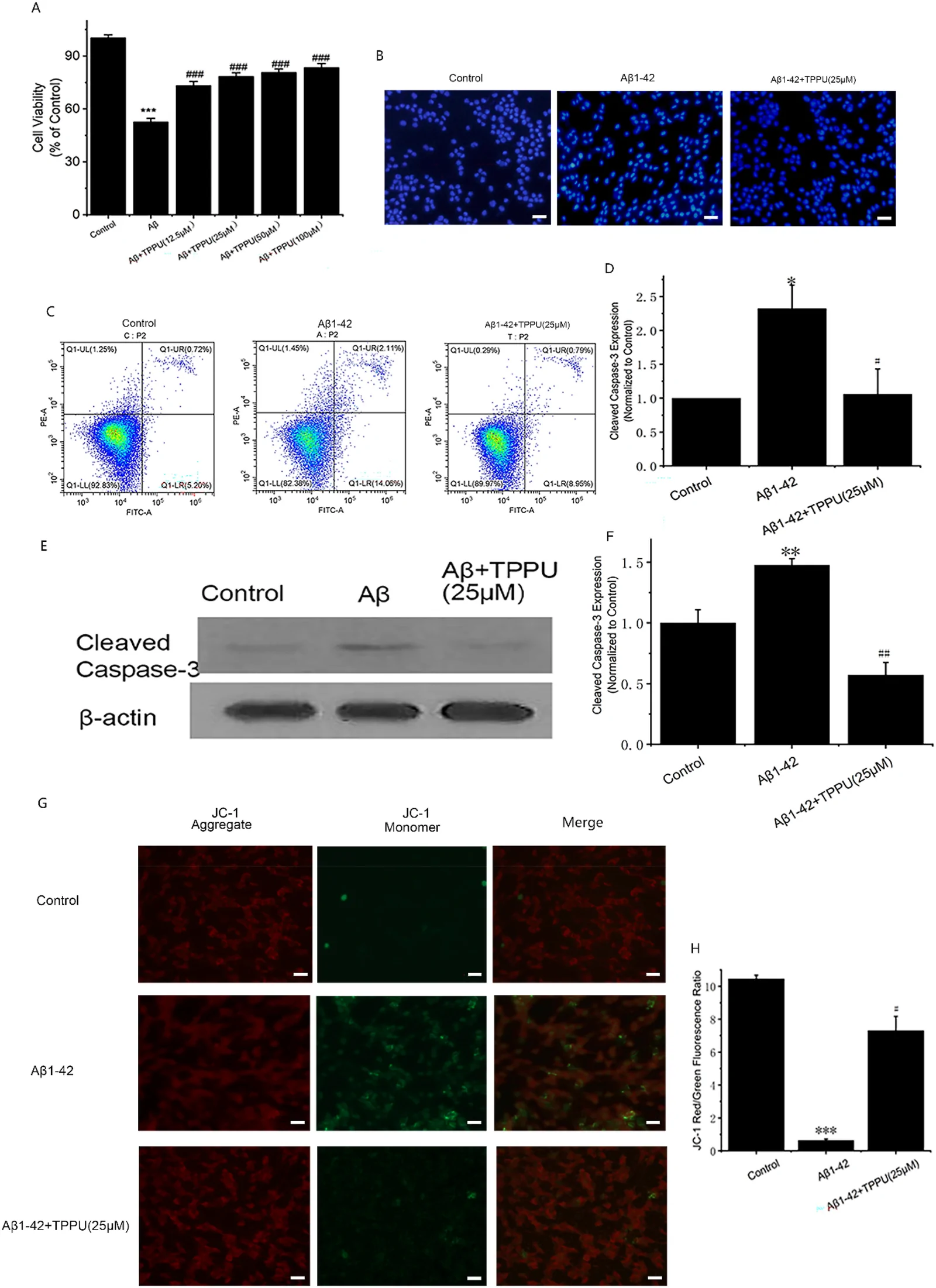
The neuroprotective effects of TPPU in Aβ1-42-treated PC-12 cells. A. The cell viability was determined using the CCK-8 assay, with results expressed as a percentage of the control (n = 55). B. The apoptotic cells were stained using Hoechst 33342 (n = 3). C. Apoptosis was analyzed by flow cytometry. D. Quantification of the apoptosis rate was performed using flow cytometry (n = 3). E. Western blot analysis of Cleaved Caspase-3 expression. F. Quantification of Cleaved Caspase-3 expression by Western blotting (n = 4). G. JC-1 staining facilitated the evaluation of mitochondrial membrane potential. H. Quantification of JC-1 rad/green fluorescence ratio (n = 3). Scale bar = 100 μm. Data = mean ± SEM. *p < 0.05 compared to control. ***p < 0.001 compared to control. #p < 0.05 compared to Aβ1-42. ###p < 0.001 compared to Aβ1-42.

### 3.2 Neuroprotective effects of the sEH inhibitor, TPPU, on cell viability and apoptosis

The neuroprotective effects of the sEH inhibitor, TPPU, were evaluated using a CCK-8 assay. Results, expressed as percentage cell viability relative to control, demonstrated that Aβ1-42 (50 μM) significantly reduced cell viability (η^2^ = 0.43; P = 0.000), confirming its neurotoxic effect on PC-12 cells. By contrast, pre-treatment with TPPU (12.5 to 100 μM) significantly improved cell viability (η^2^ = 0.51), with 12.5 μM (P = 0.000), 25 μM (P = 0.000), 50 μM (P = 0.000) and 100 μM (P = 0.000) compared with the Aβ1-42-only group (Fig. 2A). To verify that 25 μM TPPU itself is not toxic to PC-12 cells, cells were treated with TPPU at concentrations ranging from 3.1 to 100 μM for 24 h, and cell viability was measured by CCK-8 assay. As shown in Supplementary Fig. S4, TPPU at 50 μM and all lower concentrations had no significant effect on viability compared to the control; only 100 μM caused a mild reduction (η^2^ = 0.23; P = 0.000). Hoechst 33342 staining revealed prominent chromatin condensation and nuclear fragmentation in the Aβ-treated group, which was indicative of apoptosis. However, pre-treatment with TPPU markedly reduced these apoptotic features (Fig. 2B). Aβ1-42 exposure increased apoptotic rates from 4.52% to 14.95% (η^2^ = 0.79; P = 0.001), whereas TPPU reduced apoptosis to 9.82% (P = 0.019; Fig. 2C and D). These results demonstrated the anti-apoptotic effects of TPPU. Aβ1-42 (50 μM) treatment significantly increased the expression of cleaved Caspase-3 (η^2^ = 0.84; P = 0.005), indicating activation of apoptotic pathways. Notably, pre-treatment with 25 μM TPPU markedly attenuated this effect, reducing cleaved Caspase-3 levels (P = 0.001; Fig. 2E and F). Moreover, MMP was assessed using JC-1 staining. In control cells, the intensity of red fluorescence was high, while the intensity of green fluorescence was weak. Aβ1-42 (50 μM) treatment led to an increase in the green monomer form of JC-1 and a reduction in red JC-1 aggregates (η^2^ = 0.97; P < 0.001), indicating a decrease in MMP and mitochondrial dysfunction. Pre-treatment with TPPU (25 μM) reversed these effects, restoring red JC-1 aggregates and reducing green monomer fluorescence (η^2^ = 0.97; P < 0.001; Fig. 2G and H).

### 3.3 EETs improve cell viability and reduce cell death in Aβ1-42-treated PC-12 cells

EETs are substrates of sEH. In the present study, the potential effects of 1 μM 5,6-EET, 8,9-EET, 11,12-EET and 14,15-EET on cell viability and apoptosis were assessed in the Aβ-induced PC-12 model. Results of the CCK-8 assay indicated that treatment with 8,9-EET, 11,12-EET and 14,15-EET significantly enhanced cell viability under Aβ1-42 toxicity [η^2^ = 0.53; 8,9-EET (P = 0.022), 11,12-EET (P = 0.044), 14,15-EET (P < 0.001), Fig. 3A]. Results of the Hoechst 33342 staining assay revealed that apoptosis was reduced compared with the Aβ1-42-only group (Fig. 3B). These results were also confirmed using flow cytometry analysis (η^2^ = 0.52): 5,6-EET (P = 0.047), 8,9-EET (P = 0.013), 11,12-EET (P = 0.017) and 14,15-EET (P = 0.020) (Fig. 3C and D). Moreover, results of the present study revealed that MMP was also increased under these conditions (Fig. 3E and F). Exogenous 8,9-EET (P < 0.001), 11,12-EET (P = 0.011) and 14,15-EET (P = 0.004) attenuated Aβ1-42 toxicity, partially recapitulating the neuroprotective effects of TPPU through mitochondrial stabilization, and co-treatment with TPPU and each EET isoform further enhanced protection (Supplementary Fig. S3).

**Fig. 3:**
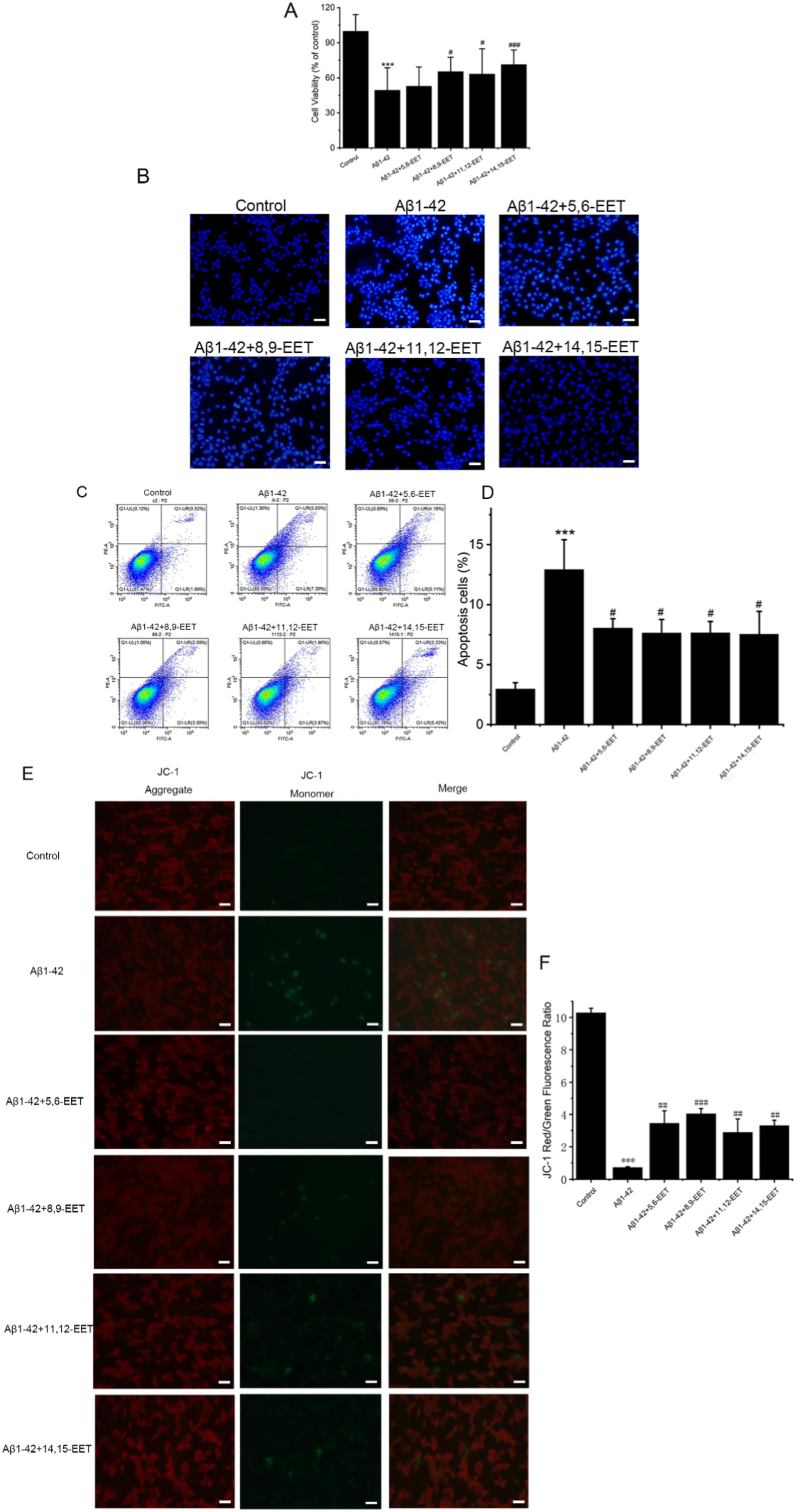
Protective effects of EETs on Aβ1-42-induced toxicity in PC-12 cells. A. Cell viability was assessed using the CCK-8 assay (n = 18). B. Apoptotic cells were stained with Hoechst 33342 (n = 3). C. Apoptosis was analyzed by flow cytometry. D. Quantification of the apoptosis rate was performed using flow cytometry (n = 3). E. Mitochondrial membrane potential was assessed with JC-1 staining. F. Quantification of JC-1 rad/green fluorescence ratio (n = 3). Scale bar = 100 μm. Data = mean ± SEM. *p < 0.05 compared to control. ***p < 0.001 compared to control. #p < 0.05 compared to Aβ1-42. ##p < 0.01 compared to Aβ1-42. ###p < 0.001 compared to Aβ1-42.

### 3.4 Regulation of JNK activation through sEH inhibition

JNK activation was investigated to determine whether the specific role of sEH inhibition in Aβ-treated PC-12 cells. Results of the Western blot analysis revealed that total JNK levels did not change significantly across groups (η^2^ = 0.37; P = 0.10; Fig. 4A and B). However, Aβ treatment significantly increased the p-JNK expression (η^2^ = 0.58; P = 0.009) (Fig. 4A and C) and the p-JNK/JNK ratio (η^2^ = 0.48; P = 0.032) (Fig. 4A and D). Compared with the Aβ group, the Aβ + TPPU group showed a significant reduction in p-JNK levels (P = 0.024) and in the p-JNK/JNK ratio (P = 0.036). These results indicate that TPPU selectively modulates JNK activation rather than total protein abundance.

**Fig. 4:**
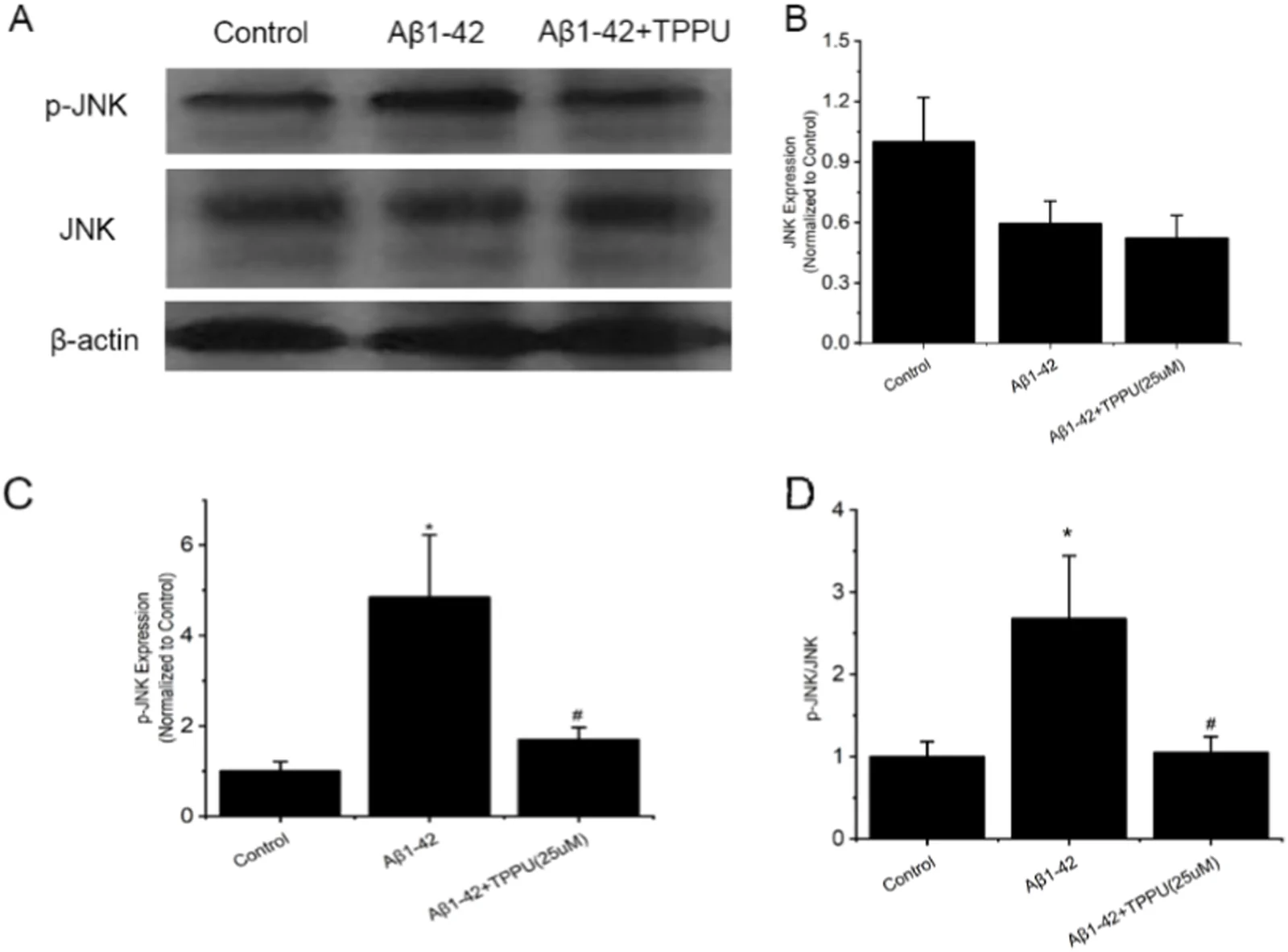
Regulation of JNK activation by TPPU. A. Western blot analysis of p-JNK and JNK expression. B. Quantification of JNK expression by Western blotting (n = 4). C. Quantification of p-JNK expression by Western blotting. D. Quantification of p-JNK/JNK expression by Western blotting (n = 4). Scale bar = 100 μm. Data = mean ± SEM. *p < 0.05 compared to control. #p < 0.05 compared to Aβ1-42.

### 3.5 Anti-apoptotic effect of sEH inhibition is comparable with that of JNK inhibition

The effects of TPPU (100 μM) and the JNK inhibitor, SP600125, were compared to investigate the mechanisms underlying the anti-apoptotic effects of sEH inhibition. Results of the CCK-8 assay revealed that both inhibitors exerted comparable protective effects (η^2^ = 0.43; Aβ + TPPU, P = 0.000; Aβ + SP600125, P = 0.000; Fig. 5A). Hoechst 33342 staining (Fig. 5B), flow cytometry (η^2^ = 0.83; Aβ + TPPU, P = 0.000; Aβ + SP600125, P < 0.001; Fig. 5C and D), and JC-1 staining for MMP (Fig. 5E) suggested that convergent pathways may mediate the observed neuroprotection (η^2^ = 0.99; Aβ + TPPU, P < 0.001; Aβ + SP600125, P < 0.001; Fig. 5E and F).

**Fig. 5:**
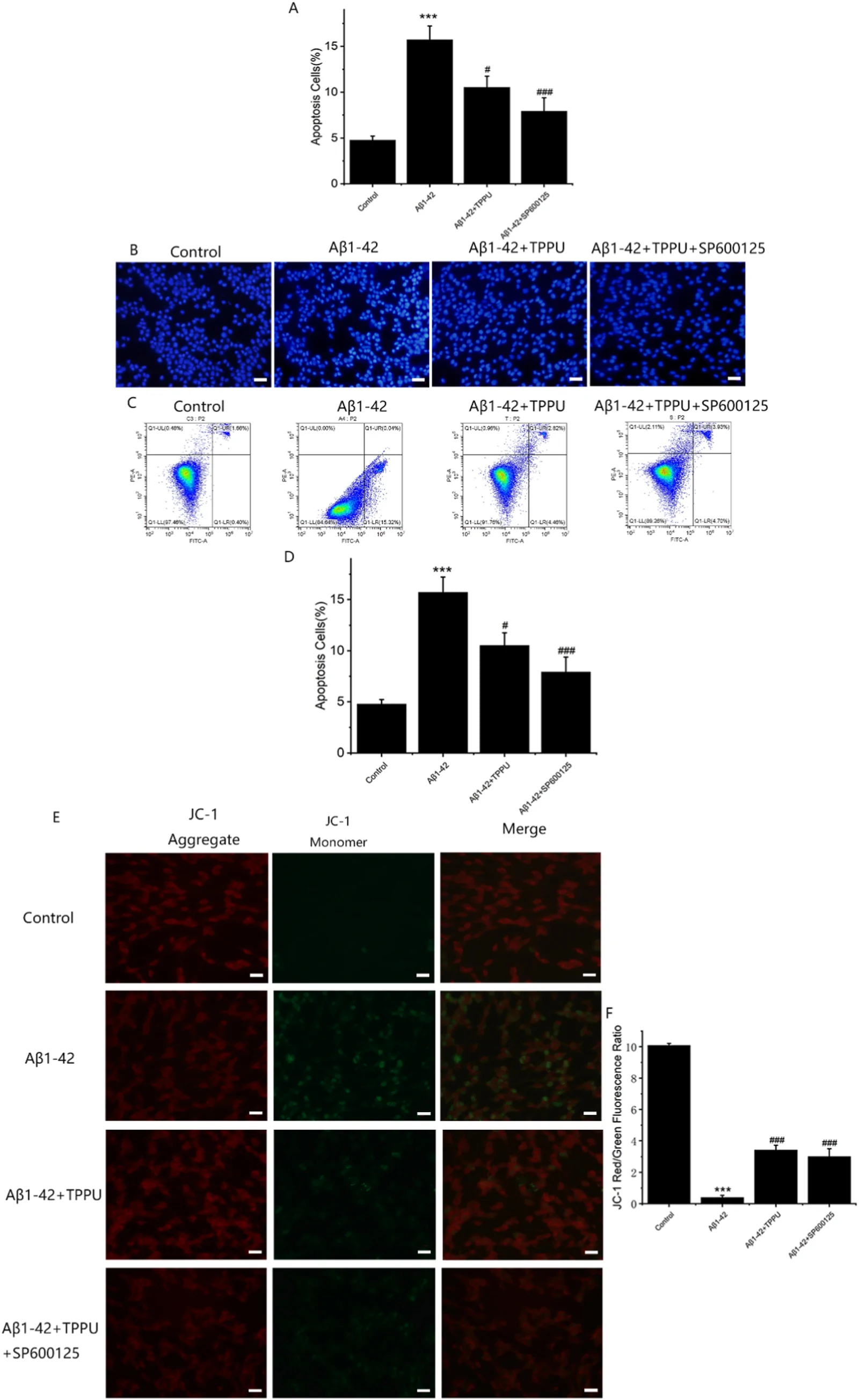
Protective effects of inhibitors on Aβ1-42-induced toxicity in PC-12 cells. A. Cell viability was assessed using the CCK-8 assay (n = 18). B. Apoptotic cells were stained with Hoechst 33342 (n = 3). C. Apoptosis was analyzed by flow cytometry. D. Quantification of apoptosis rate using flow cytometry (n = 3). E. JC-1 staining was used to assess mitochondrial membrane potential. F. Quantification of JC-1 rad/green fluorescence ratio (n = 3). Scale bar = 100 μm. Data = mean ± SEM. ***p < 0.001 compared to control. #p < 0.05 compared to Aβ1-42. ###p < 0.001 compared to Aβ1-42.

## 4 Discussion

AD is a progressive neurodegenerative disorder characterized by Aβ plaque deposition, neurofibrillary tangles and synaptic loss, ultimately leading to neuronal death [1]. Despite extensive research into AD pathogenesis, the development of disease-modifying therapies remains challenging [4]. Among several potential neuroprotective targets, sEH has been proposed to exert beneficial effects [12]. Pharmacological inhibition of sEH has demonstrated neuroprotective potential in preclinical models of neurodegeneration [25]; thus, the present study aimed to evaluate the effects of sEH inhibition on Aβ-induced cytotoxicity in PC-12 cells. Results of the present study revealed that TPPU, a potent sEH inhibitor, reduced Aβ1-42-induced neurotoxicity via suppression of the JNK pathway. These results highlighted that TPPU-mediated inhibition of sEH significantly mitigated Aβ-induced neuronal damage. A notable increase in cell viability and a substantial reduction in cell death were observed after administering TPPU to Aβ-treated PC-12 cells. In addition, sEH inhibition reversed apoptotic features induced by Aβ, such as chromatin condensation, nuclear fragmentation and mitochondrial dysfunction. These findings suggested that sEH inhibition may regulate critical signaling pathways associated with cell survival. Specifically, TPPU treatment regulates JNK activation, a pathway involved in inflammation and apoptosis. Notably, results of the present study highlighted a potential dual-pathway neuroprotective mechanism (Fig. 6), in which TPPU preserves MMP and suppresses Bax/Bak pore formation. These findings are comparable with those of a previous study [32]. Thus, sEH inhibition may enhance neuroprotection through the elevation of EET levels, which, in turn, activate signaling for cell survival, such as mitochondrial function restoration and JNK suppression. The co-treatment experiment further showed that each of the four EET isoforms significantly enhanced the protective effect of TPPU, supporting the idea that the sEH-EET axis contributes positively to TPPU's neuroprotection.

**Fig. 6:**
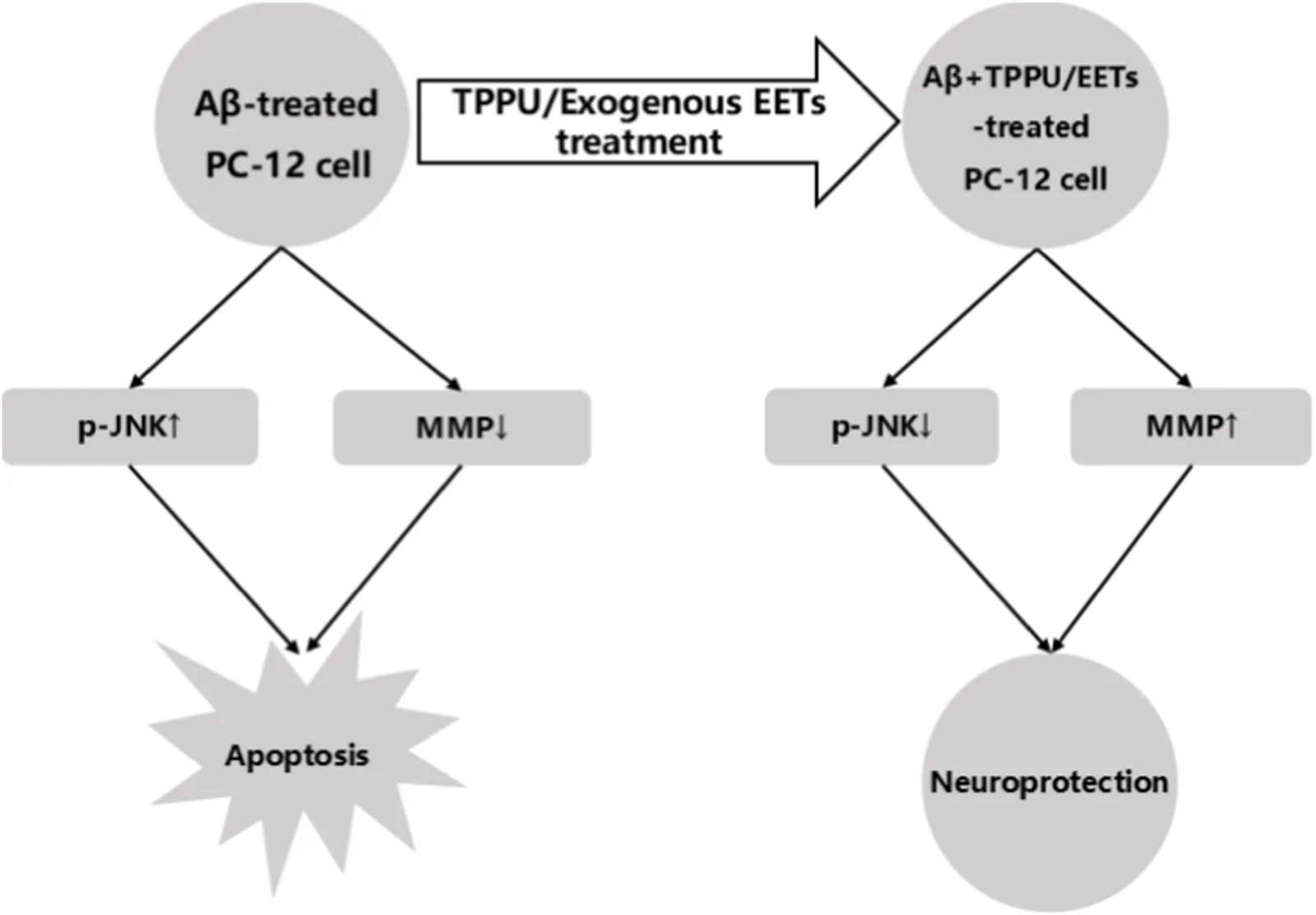
Schematic diagram illustrating the proposed dual neuroprotective mechanisms of TPPU against Aβ-induced toxicity in PC-12 cells. In Aβ-treated cells, mitochondrial dysfunction and JNK activation are two parallel pathological events that converge to trigger neuronal apoptosis. Upon TPPU/exogenous EETs treatment, both events are simultaneously counteracted: mitochondrial membrane potential is restored and JNK phosphorylation is suppressed. This concurrent action diverts the cellular outcome from apoptosis toward neuroprotection, highlighting the dual mechanism of TPPU.

Numerous previous studies have implicated sEH in various pathologies [20,21,33]. The present study provides complementary mechanistic evidence, as the results demonstrated that sEH inhibition protects against Aβ-induced neurotoxicity in a neuronal cell model. Thus, modulation of the sEH-EET axis may ameliorate Aβ toxicity, indicating that JNK suppression and mitochondrial stabilization may act as key cellular effectors. Therefore, TPPU exhibits potential in the treatment of neurodegenerative diseases.

Collectively, results of the present study highlighted that targeting sEH may exhibit potential as a therapeutic approach for the treatment of AD. Clinically, sEH inhibitors, such as TPPU, may reduce the neurotoxic effects of Aβ. This may slow disease progression or prevent early damage associated with Aβ plaque accumulation. Given that JNK activation drives tau hyperphosphorylation [34] and sEH inhibition suppresses neuroinflammation [35], TPPU may exhibt broader therapeutic implications beyond Aβ toxicity. Future *in vivo* and clinical studies should explore the efficacy of sEH inhibitors in animal models of AD, and assess their long-term safety and effectiveness. In addition, a combination of sEH inhibition with other therapeutic approaches should be investigated to enhance neuroprotection.

Notably, the present study exhibits numerous limitations. The experiments were conducted using a cell culture model, which does not fully replicate the complex *in vivo* environment of the AD brain. In particular, all experiments were performed using the PC-12 cell line, which, despite being a well-established model for preliminary neurotoxicity screening, does not fully recapitulate the properties of primary neurons. Therefore, our findings should be validated in primary cortical or hippocampal neurons in future studies. Key limitations include the absence of crucial elements, such as blood-brain barrier interactions, systemic inflammation and the role of glial cells in neurodegeneration. Moreover, the long-term effects of sEH suppression on neuronal survival and cognitive function remain to be fully elucidated. Future research should involve animal models of AD to establish the therapeutic potential of sEH inhibition *in vivo*. In addition, the optimal dosage and delivery mechanisms for sEH inhibitors should also be evaluated to validate their efficacy while minimizing potential side effects. In addition, a dedicated positive control for phosphorylated JNK was not included in our Western blot analysis. The untreated control group served as a baseline reference, but we acknowledge that an induced positive control would further validate antibody specificity. This remains a limitation to be addressed in future studies. We also did not test the diol metabolites (DHETs) because they are generally less bioactive and were not available for this study; this remains a limitation to be addressed in future work.

Future investigations should focus on determining the specific molecular mechanisms underlying the protective effects of sEH inhibition on Aβ toxicity. Proteomic analyses are required to identify additional signaling pathways modulated by TPPU, and to determine interactions with other cellular processes associated with AD pathology. Additional studies should also explore the impact of combining sEH inhibitors with other neuroprotective agents. This combinatorial approach may improve therapeutic outcomes through simultaneously targeting multiple aspects of AD pathology. The development of more selective sEH inhibitors with enhanced pharmacokinetic properties is also required, to improve blood-brain barrier penetration.

## 5 Conclusions

The present study highlighted that sEH inhibition may mitigate Aβ-induced neuronal damage in PC-12 cells. To the best of our knowledge, the present study is the first to demonstrate the potential association between sEH and Aβ-specific JNK dysregulation. sEH inhibitors, such as TPPU, may exhibit potential as a therapeutic strategy for AD; however, further validation in animal models and in clinical settings is required. Further understanding the neuroprotective effects of sEH inhibition may improve outcomes for patients with AD.

## Ethics statement

This study did not involve human subjects or human samples. All experiments were conducted in accordance with relevant institutional guidelines.

## Author agreement

All authors have read and approved the final version of the manuscript. The work described has not been published previously and is not under consideration for publication elsewhere. All authors agree to its submission to Biochemistry and Biophysics Reports.

## Author contribution

QW, J-F S performed the experiments and analyzed the data. QW drafted the manuscript. J-F S, A-L H and S-Y L designed the experiments and supervised the project. All authors were involved in data quantification and contributed to the manuscript revision.

## Funding

The present study was supported by the 2024 Guangdong Medical Research Fund Project (grant no. A2024632), 2021 Jiangmen Basic and Application Basic Key Project (17) and Research, Innovation team of Guangdong Jiangmen Vocational College of Traditional Chinese Medicine (grant no. JMZYYKCT20233001) and Key Platform Project of the Department of Education of Guangdong Province (grant no. 2022GCZX023).

## Declaration of competing interest

The authors declare that they have no known competing financial interests or personal relationships that could have appeared to influence the work reported in this paper.

## Data Availability

Data will be made available on request.

## References

[1] d'Errico P., Meyer-Luehmann M. (2020). Mechanisms of pathogenic tau and abeta protein spreading in Alzheimer's disease. Front. Aging Neurosci..

[2] Paroni G., Bisceglia P., Seripa D. (2019). Understanding the amyloid hypothesis in Alzheimer's disease. J. Alzheimers Dis..

[3] Chen G.F., Xu T.H., Yan Y., Zhou Y.R., Jiang Y., Melcher K., Xu H.E. (2017). Amyloid beta: structure, biology and structure-based therapeutic development. Acta Pharmacol. Sin..

[4] Weller J., Budson A. (2018). Current understanding of Alzheimer's disease diagnosis and treatment. F1000Res.

[5] Masters C.L., Bateman R., Blennow K., Rowe C.C., Sperling R.A., Cummings J.L. (2015). Alzheimer's disease. Nat. Rev. Dis. Primers.

[6] Walker L.C. (2020). Abeta plaques. Free Neuropathol. 1.

[7] Tolar M., Hey J., Power A., Abushakra S. (2021). Neurotoxic soluble amyloid oligomers drive alzheimer's pathogenesis and represent a clinically validated target for slowing disease progression. Int. J. Mol. Sci..

[8] Marttinen M., Takalo M., Natunen T., Wittrahm R., Gabbouj S., Kemppainen S., Leinonen V., Tanila H., Haapasalo A., Hiltunen M. (2018). Molecular mechanisms of synaptotoxicity and neuroinflammation in Alzheimer's disease. Front. Neurosci..

[9] Fantini J., Chahinian H., Yahi N. (2020). Progress toward Alzheimer's disease treatment: leveraging the Achilles' heel of Abeta oligomers?. Protein Sci..

[10] Pal C. (2025). Mitochondria-targeting by small molecules against Alzheimer's disease: a mechanistic perspective. Biochim. Biophys. Acta Mol. Basis Dis..

[11] Choi S.B., Kwon S., Kim J.H., Ahn N.H., Lee J.H., Yang S.H. (2023). The molecular mechanisms of neuroinflammation in Alzheimer's disease, the consequence of neural cell death. Int. J. Mol. Sci..

[12] Li Y., Sun Z., Cao Q., Chen M., Luo H., Lin X., Xiao F. (2017). Role of amyloid beta protein receptors in mediating synaptic plasticity. Biomed. Rep..

[13] Xia N., Xu L., Xu D., Huang M., Li Y., Mei Z., Yu Z. (2024). Neuroprotective effect of emodin on Abeta(25-35)-induced cytotoxicity in PC12 cells involves Nrf2/GPX4 and TLR4/p-NF-kappaB/NLRP3 pathways. Brain Res..

[14] Nelson J.W., Young J.M., Borkar R.N., Woltjer R.L., Quinn J.F., Silbert L.C., Grafe M.R., Alkayed N.J. (2014). Role of soluble epoxide hydrolase in age-related vascular cognitive decline. Prostag. Other Lipid Mediat..

[15] Wagner K.M., McReynolds C.B., Schmidt W.K., Hammock B.D. (2017). Soluble epoxide hydrolase as a therapeutic target for pain, inflammatory and neurodegenerative diseases. Pharmacol. Ther..

[16] Wu Y., Dong J.H., Dai Y.F., Zhu M.Z., Wang M.Y., Zhang Y., Pan Y.D., Yuan X.R., Guo Z.X., Wang C.X., Li Y.Q., Zhu X.H. (2023). Hepatic soluble epoxide hydrolase activity regulates cerebral Abeta metabolism and the pathogenesis of Alzheimer's disease in mice. Neuron.

[17] Fromel T., Hu J., Fleming I. (2023). Lipid mediators generated by the cytochrome P450-Epoxide hydrolase pathway. Adv. Pharmacol..

[18] Grinan-Ferre C., Jarne-Ferrer J., Bellver-Sanchis A., Codony S., Puigoriol-Illamola D., Sanfeliu C., Oh Y., Lee S., Vazquez S., Pallas M. (2024). Novel molecular mechanism driving neuroprotection after soluble epoxide hydrolase inhibition: insights for Alzheimer's disease therapeutics. CNS Neurosci. Ther..

[19] Ghosh A., Comerota M.M., Wan D., Chen F., Propson N.E., Hwang S.H., Hammock B.D., Zheng H. (2020). An epoxide hydrolase inhibitor reduces neuroinflammation in a mouse model of Alzheimer's disease. Sci. Transl. Med..

[20] Kuo Y.M., Lee Y.H. (2022). Epoxyeicosatrienoic acids and soluble epoxide hydrolase in physiology and diseases of the central nervous system. Chin. J. Physiol..

[21] Domingues M.F., Callai-Silva N., Piovesan A.R., Carlini C.R. (2019). Soluble epoxide hydrolase and brain cholesterol metabolism. Front. Mol. Neurosci..

[22] Zhao Y., Kuca K., Wu W., Wang X., Nepovimova E., Musilek K., Wu Q. (2022). Hypothesis: JNK signaling is a therapeutic target of neurodegenerative diseases. Alzheimer's Dement..

[23] Solas M., Vela S., Smerdou C., Martisova E., Martinez-Valbuena I., Luquin M.R., Ramirez M.J. (2023). JNK activation in Alzheimer's disease is driven by amyloid beta and is associated with Tau pathology. ACS Chem. Neurosci..

[24] Okazawa H., Estus S. (2002). The JNK/c-Jun cascade and Alzheimer's disease. Am. J. Alzheimers Dis. Other Demen..

[25] Mehan S., Meena H., Sharma D., Sankhla R. (2011). JNK: a stress-activated protein kinase therapeutic strategies and involvement in Alzheimer's and various neurodegenerative abnormalities. J. Mol. Neurosci..

[26] Xu J., Qin X., Cai X., Yang L., Xing Y., Li J., Zhang L., Tang Y., Liu J., Zhang X., Gao F. (2015). Mitochondrial JNK activation triggers autophagy and apoptosis and aggravates myocardial injury following ischemia/reperfusion. Biochim. Biophys. Acta.

[27] Dhanasekaran D.N., Reddy E.P. (2008). JNK signaling in apoptosis. Oncogene.

[28] Putcha G.V., Le S., Frank S., Besirli C.G., Clark K., Chu B., Alix S., Youle R.J., LaMarche A., Maroney A.C., Johnson E.M. (2003). JNK-mediated BIM phosphorylation potentiates BAX-dependent apoptosis. Neuron.

[29] Waetzig V., Herdegen T. (2004). Neurodegenerative and physiological actions of c-Jun N-terminal kinases in the mammalian brain. Neurosci. Lett..

[30] Wang M.J., Jeng K.C., Kuo J.S., Chen H.L., Huang H.Y., Chen W.F., Lin S.Z. (2004). c-Jun N-terminal kinase and, to a lesser extent, p38 mitogen-activated protein kinase regulate inducible nitric oxide synthase expression in hyaluronan fragments-stimulated BV-2 microglia. J. Neuroimmunol..

[31] Kang C.D., Jang J.H., Kim K.W., Lee H.J., Jeong C.S., Kim C.M., Kim S.H., Chung B.S. (1998). Activation of c-jun N-terminal kinase/stress-activated protein kinase and the decreased ratio of Bcl-2 to Bax are associated with the auto-oxidized dopamine-induced apoptosis in PC12 cells. Neurosci. Lett..

[32] Yi X., Fan D., Yi T., Chen H., Qing T., Han Z., Bao S. (2021). 1-trifluoromethoxyphenyl-3-(1-propionylpiperidin-4-yl) urea exerts neuro-protective effects against ischemic injury via suppressing JNK/p38 MAPK-mediated mitochondrial apoptosis pathway. J. Stroke Cerebrovasc. Dis..

[33] Noh M.R., Jang H.S., Salem F.E., Ferrer F.A., Kim J., Padanilam B.J. (2023). Epoxyeicosatrienoic acid administration or soluble epoxide hydrolase inhibition attenuates renal fibrogenesis in obstructive nephropathy. Am. J. Physiol. Ren. Physiol..

[34] Coffey E.T. (2014). Nuclear and cytosolic JNK signalling in neurons. Nat. Rev. Neurosci..

[35] Fang X., Border J.J., Zhang H., Challagundla L., Kaur J., Hwang S.H., Hammock B.D., Fan F., Roman R.J. (2025). A soluble epoxide hydrolase inhibitor improves cerebrovascular dysfunction, neuroinflammation, amyloid burden, and cognitive impairments in the hAPP/PS1 TgF344-AD rat model of Alzheimer's disease. Int. J. Mol. Sci..

