# Supplementary material for: TPPU, a soluble epoxide hydrolase inhibitor, mitigates Aβ-induced neurotoxicity via dual modulation of mitochondrial stabilization and JNK suppression
Source: Biochem Biophys Rep. 2026 Oct 12;48:102806. doi: 10.1016/j.bbrep.2026.102806 (PMC13681467; doi:10.1016/j.bbrep.2026.102806)
Supplement: Supplementary file 1 [file mmc1.docx]

Supplementary Fig. S1. Uncropped original Western blot membranes for sEH, JNK, GAPDH and cleaved Caspase-3. Each membrane includes the Control, Aβ1-42 and TPPU lanes. Molecular weight markers are indicated on the left.

Supplementary Figure S2. Uncropped original Western blot membranes for p‑JNK.

Each membrane includes the Control, Aβ1-42 and TPPU lanes. Molecular weight markers are indicated on the left.

Supplementary Fig. S3. Effect of co-treatment with TPPU and EETs on Aβ-induced cytotoxicity. PC-12 cells were treated as indicated. Cell viability was measured by CCK-8 assay. Data are mean ± SD (n = 18 per group). ***P < 0.001 vs. Aβ; #P < 0.05, ##P < 0.01, ###P < 0.001 vs. TPPU.

Supplementary Fig. S4. Effect of TPPU alone on PC-12 cell viability. Cells were treated with indicated concentrations of TPPU for 24 h, and cell viability was measured by CCK-8 assay. Data are mean ± SD. ***P < 0.001 vs. control.
